# Effect of Au Film Thickness and Surface Roughness on Room-Temperature Wafer Bonding and Wafer-Scale Vacuum Sealing by Au-Au Surface Activated Bonding

**DOI:** 10.3390/mi11050454

**Published:** 2020-04-27

**Authors:** Michitaka Yamamoto, Takashi Matsumae, Yuichi Kurashima, Hideki Takagi, Tadatomo Suga, Seiichi Takamatsu, Toshihiro Itoh, Eiji Higurashi

**Affiliations:** 1 The University of Tokyo, 5-1-5 Kashiwanoha, Kashiwa-shi, Chiba 277-8563, Japan; myamamoto@s.h.k.u-tokyo.ac.jp (M.Y.); seiichi-takamatsu@edu.k.u-tokyo.ac.jp (S.T.); toshihiro-itoh@edu.k.u-tokyo.ac.jp (T.I.); 2 National Institute of Advanced Industrial Science and Technology (AIST), 1-2-1 Namiki, Tsukuba, Ibaraki 305-8564, Japan; t.matsumae@aist.go.jp (T.M.); y-kurashima@aist.go.jp (Y.K.); takagi.hideki@aist.go.jp (H.T.); 3 Meisei University, 2-1-1 Hodokubo, Hino, Tokyo 191-8506, Japan; suga@gakushikai.jp

**Keywords:** heterogeneous integration, wafer bonding, wafer sealing, room-temperature bonding, Au-Au bonding, surface activated bonding, Au film thickness, surface roughness

## Abstract

Au-Au surface activated bonding (SAB) using ultrathin Au films is effective for room-temperature pressureless wafer bonding. This paper reports the effect of the film thickness (15–500 nm) and surface roughness (0.3–1.6 nm) on room-temperature pressureless wafer bonding and sealing. The root-mean-square surface roughness and grain size of sputtered Au thin films on Si and glass wafers increased with the film thickness. The bonded area was more than 85% of the total wafer area when the film thickness was 100 nm or less and decreased as the thickness increased. Room-temperature wafer-scale vacuum sealing was achieved when Au thin films with a thickness of 50 nm or less were used. These results suggest that Au-Au SAB using ultrathin Au films is useful in achieving room-temperature wafer-level hermetic and vacuum packaging of microelectromechanical systems and optoelectronic devices.

## 1. Introduction

Sealing techniques are essential to protect the sensitive elements of microelectromechanical systems (MEMS) and optoelectronic devices from the environment [1,2,3]. An effective way to achieve sealing is to bond cap wafers to device wafers. Many types of bonding techniques such as anodic bonding [4], thermocompression bonding [5,6,7], solder bonding [8,9], and eutectic bonding [10] have been used as sealing techniques. However, these techniques require high bonding temperature, which causes problems such as thermally induced mechanical stress due to thermal expansion mismatch. Therefore, low-temperature bonding using metal intermediate layers is becoming increasingly attractive because of the high bonding strength and good reliability that can now be achieved. Research on bonding using Au intermediate layers has been increasing [11,12,13,14,15,16,17,18,19,20,21,22,23,24,25,26,27,28,29,30,31,32,33,34,35], because Au has several highly desirable properties such as high resistance to oxidation and corrosion.

Au-Au surface activated bonding (SAB) [17,18,19,20,21,22,23,24,25,26,27,28,29,30,31,32,33,34,35] is a promising technique for low-temperature bonding. In Au-Au SAB, the Au surfaces are activated by plasma treatment and then brought into contact at low temperature (<150 °C). Au-Au SAB has also been applied to hermetic sealing, as well as the integration of different materials [17,24]. An advantage of Au-Au SAB is that the Au films can be patterned using photolithography before bonding, enabling high transparency to be achieved by using glass wafers [35]. For example, chip-scale hermetic sealing in air has been achieved at low temperature (150 °C). However, high bonding pressure (300 MPa) was necessary because thick Au films (thickness: 300–500 nm) with rough surfaces (root mean square (RMS) surface roughness: 4.0 nm) were used as sealing rings [24]. Various methods such as thermal-imprint [25], lift-off [26], and direct-transfer [31] have been investigated to reduce the bonding pressure required for sealing by using Au-Au SAB. However, high bonding pressure (>100 MPa) is still required to achieve sealing [28].

Room-temperature pressureless wafer bonding was recently achieved with Au-Au SAB using ultrathin Au films (thickness <50 nm) with small grains, and thus, smooth surfaces (RMS surface roughness: <0.5 nm) [30,34]. Furthermore, room-temperature pressureless wafer-scale hermetic sealing in both air and vacuum was achieved using Au-Au SAB with ultrathin Au films (thickness: 15 nm) [35]. However, the effect of the film thickness on Au-Au bondability and sealing quality has not been investigated quantitatively.

This paper reports on the use of Au thin films with different film thicknesses in room-temperature pressureless wafer bonding and vacuum sealing processes. We also investigate the effect of film thickness and surface roughness on wafer bonding and vacuum sealing quality.

## 2. Experimental Methods

### 2.1. Room-Temperature Pressureless Wafer Bonding in Ambient Air

In the first experiment, Au thin films with different thicknesses (15, 50, 100, 300, 500 nm) and Ti thin films with a thickness of 5 nm as adhesion layers were deposited on 4-inch Si wafers by DC sputtering (JSP-8000, ULVAC, Inc., Chigasaki, Japan). The sputtering was performed at a chamber pressure of 0.15 Pa and a sputtering power of 200 W for the Ti films and 100 W for the Au films. The surface roughness of the deposited films was measured with an atomic force microscope (AFM; L-trace, Hitachi High-Tech Science Corporation, Tokyo, Japan), with a scanning area of 500 nm × 500 nm. The average grain size of the Au films was calculated from the observed AFM data using the watershed algorithm [36]. To investigate the stress in the deposited films, we measured the curvature radius of the wafers before and after film deposition using a thin-film stress measurement system (FLX-2320-S, TOHO Inc., Nagoya, Japan). The film stress *σ_f_* was calculated using the Stoney equation [37]:

(1) $$\sigma_{f}=\frac{{E_{s}t_{s}}^{2}}{6\left(1-\nu_{s}\right)t_{f}}\cdot \left(\frac{1}{R_{1}}-\frac{1}{R_{0}}\right)$$

where *E_s_*, *ν_s_*, and *t_s_*, are Young’s modulus, Poisson’s ratio, and substrate thickness, respectively, *R*_0_ and *R*_1_ are the curvature radii of the wafer before and after film deposition, and *t_f_* is the deposited film thickness. In this work, Si was assumed to be isotropic, and Young’s modulus, Poisson’s ratio, and substrate thickness were set to 169 GPa, 0.06, and 525 µm, respectively [38,39]. The *t_f_* was calculated as the total thickness of the Au and Ti thin films.

The bonding was performed by placing two wafers with the Au sides facing each other in ambient air and squeezing their centers together with tweezers once with an estimated applied force of <10 N. Before bonding, Ar plasma treatment (RF power: 200 W, operating pressure: 60 Pa, treatment time: 60 s) was performed for surface activation using the plasma equipment installed in the bonding system (WAP-1000, Bondtech Co., Ltd., Kyoto, Japan). The treatment time (60 s) was short enough not to affect the surface roughness of the Au surfaces [34]. The bonded area was observed with a surface acoustic microscope (SAM; SAM 300, PVA TePla Analytical Systems, Westhausen, Germany), and the percentage of the bonded area was calculated using ImageJ software [40]. The bonding strength was evaluated using the razor blade test, which is also known as the crack opening method [41]. The crack length caused by inserting a blade was observed with the SAM.

### 2.2. Room-Temperature Wafer Sealing in Vacuum

**Figure 1:**
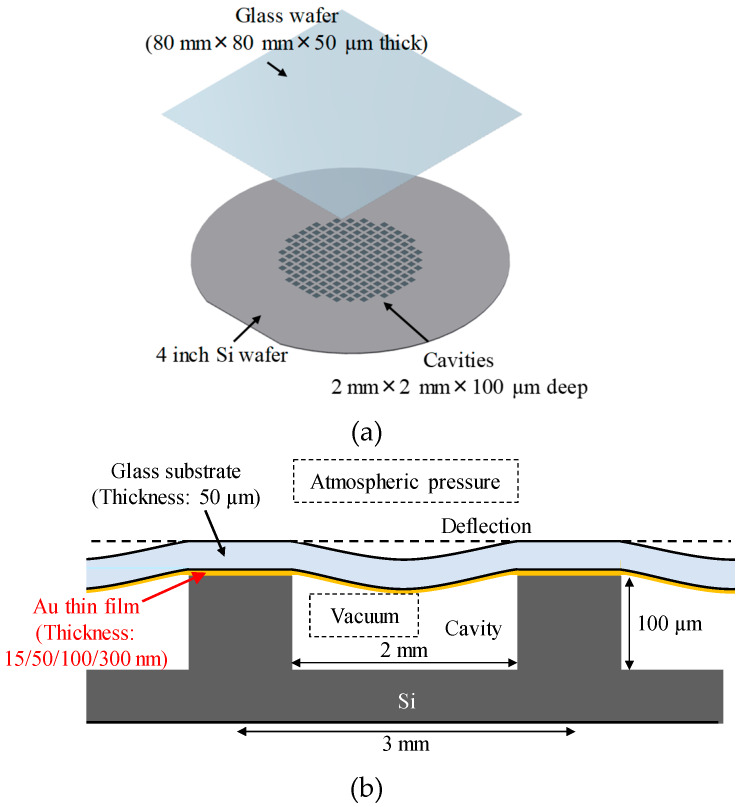
Schematics of bonded wafer pair and bonded structure: (**a**) Wafer pair (thin glass wafer and Si wafer with cavities) deposited with Au thin films (**b**) Cross-sectional schematic of vacuum-sealed sample. Glass substrate exhibited deflection due to pressure difference between sealed vacuum cavity and ambient atmosphere.

In the second experiment, Au thin films with different film thicknesses (15, 50, 100, 300 nm) and Ti thin films with a thickness of 5 nm as adhesion layers were deposited on Si wafers (4-inch diameter) with cavities and on alkali-free ultrathin glass wafers (80 mm square and 50 µm thick, G-Leaf, Nippon Electric Glass Co., Ltd., Otsu, Japan) by DC sputtering. More than 100 cavities with lateral dimensions of 2 mm × 2 mm, a depth of 100 µm, and a pitch of 3 mm were fabricated in the middle of the wafers by wet chemical etching. A schematic of a bonded wafer pair is shown in Figure 1a, and a cross-sectional schematic of a vacuum-sealed sample is shown in Figure 1b. The surface roughness of each wafer was measured with the AFM, and the average grain size was calculated using the watershed algorithm.

Room-temperature vacuum sealing was performed using the bonding system (WAP-1000, Bondtech Co., Ltd.). Two wafers were bonded in a vacuum chamber (~10^−2^ Pa) at room temperature and a contact load of 2000 N. Before bonding, the Au surfaces were activated by Ar plasma (RF power: 200 W, operating pressure: 60 Pa, treatment time: 60 s). The applied contact load (2000 N) corresponded to less than 1.6 MPa for the bonded samples.

The sealing quality of the vacuum-sealed samples was evaluated by visually checking the number of cavities with cap deflection. Since the glass wafers were thin (thickness: 50 µm), the glass caps on the vacuum-sealed cavities exhibited deflection after bonding due to the pressure difference between the sealed vacuum cavities and the ambient atmosphere, as shown in Figure 1b. Furthermore, microstructure observation of the bonded interface was performed with a transmission electron microscope (TEM; H-9500, Hitachi High-Tech Science Co., Tokyo, Japan).

## 3. Results

### 3.1. Room-Temperature Pressureless Wafer Bonding in Ambient Air

**Figure 2:**
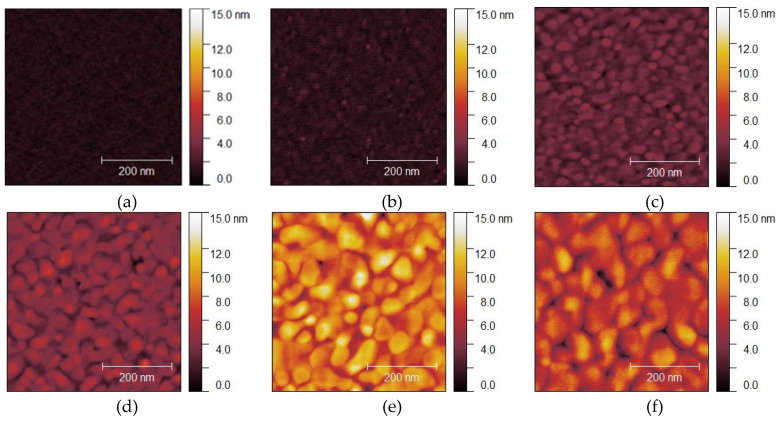
Typical atomic force microscope (AFM) images of Au thin films deposited on Si wafers: (**a**) before deposition; (**b**–**f**) deposited films with thicknesses of 15, 50, 100, 300, and 500 nm, respectively.

**Figure 3:**
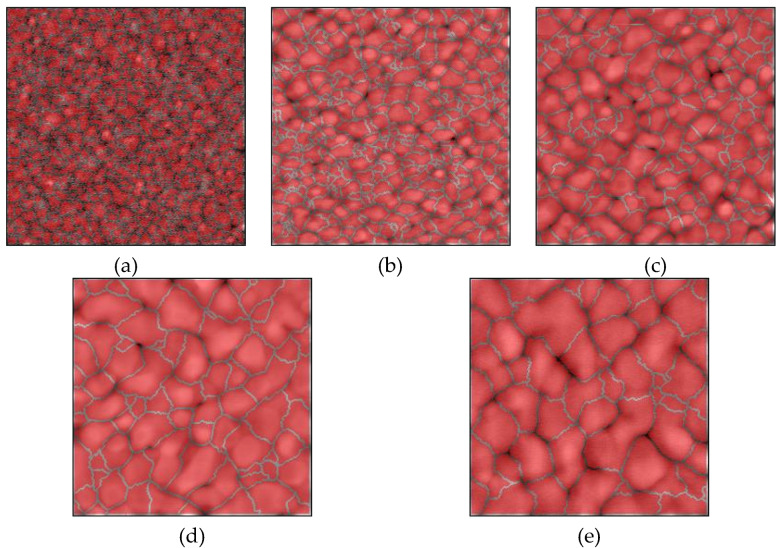
Typical AFM images of Au thin films deposited on Si wafers with grains segmented using the watershed algorithm: (**a**–**e**) films with thicknesses of 15, 50, 100, 300, and 500 nm, respectively.

**Figure 4:**
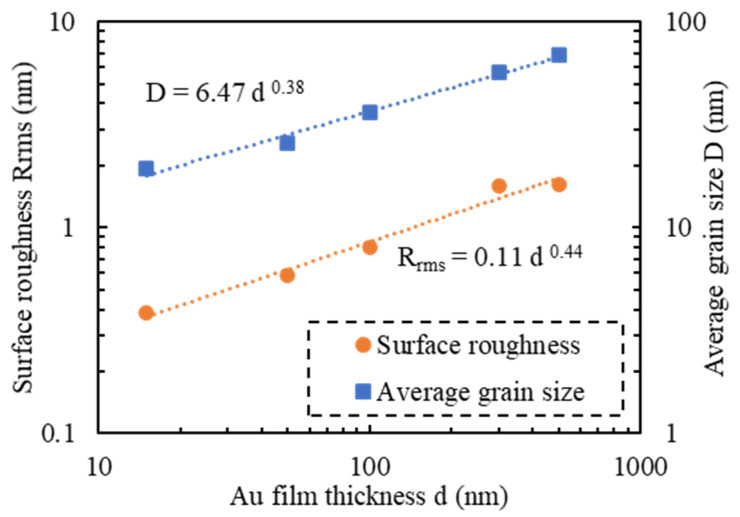
Effect of thickness of Au films deposited on Si wafers on surface roughness and average grain size.

A measured AFM image of a Si wafer before Au thin film deposition is shown in Figure 2a, and the images of Au thin films with thicknesses of 15, 50, 100, 300, and 500 nm deposited on Si wafers are shown in Figure 2b–f, respectively. Before film deposition, the RMS surface roughness was 0.3 nm. The grain geometry of each Au thin film (Figure 3) was determined using the watershed algorithm. The effect of the film thickness on the surface roughness and average grain size deposited on the Si wafers is illustrated in Figure 4. Both increased exponentially with the thickness, which is consistent with the results of previous studies [42]. 

**Figure 5:**
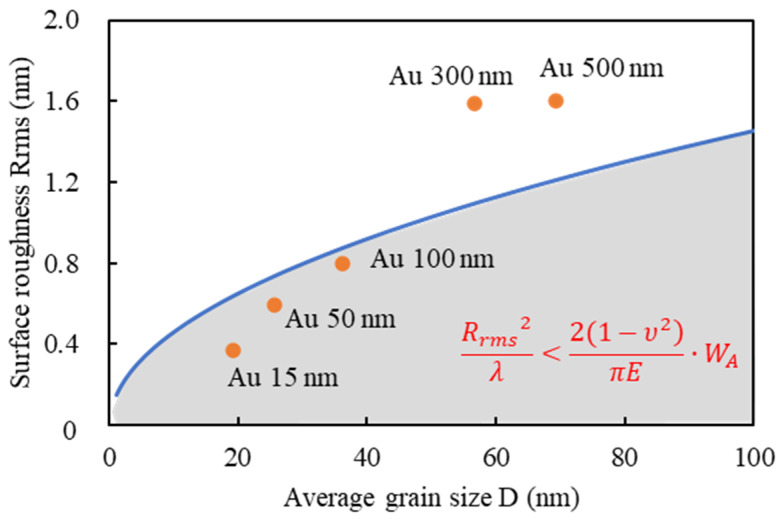
Relationship between surface roughness calculated using Equation (2) and average grain size. Measured average grain size and surface roughness of Au thin films with different thicknesses are also plotted.

In a previous study [43], the effect of surface roughness on spontaneous bonding was discussed in terms of elastic deformation and energy gain due to bond formation. If bonding is to be achieved, the elastic energy must be smaller than the work of adhesion, *W_A_*, i.e., the energy gain due to bond formation at the interface. If the surface profile is assumed to be a sinusoidal curve, the surface is assumed to be elastic, and wavelength λ is assumed to correspond to the average grain size, the necessary surface roughness for pressureless bonding can be estimated using

(2) $$\frac{{R_{rms}}^{2}}{\lambda }<\frac{2\left(1-\nu^{2}\right)}{\pi E}\cdot W_{A}$$

where *R_rms_* and *λ* are the RMS surface roughness and wavelength of the bonding surface. *E* and *ν* are Young’s modulus and Poisson’s ratio. The relationship between average grain size and surface roughness calculated with this formula is shown in Figure 5. The measured average grain size and surface roughness of Au thin films with different thicknesses are also plotted. A thickness of 100 nm or less satisfied the above assumptions, and pressureless bonding should thus be achieved.

**Figure 6:**
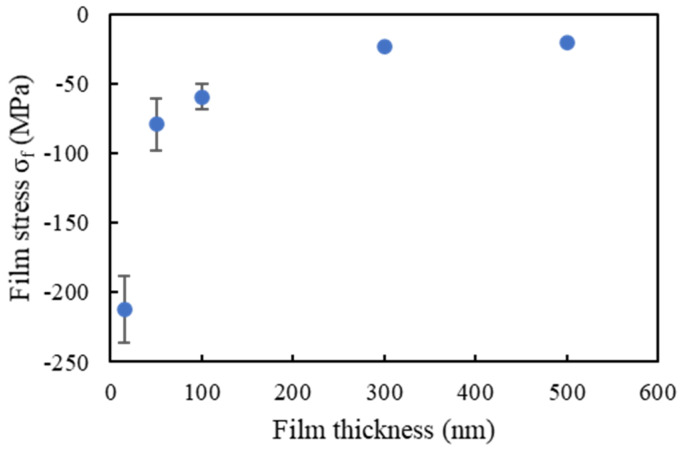
Residual stress in Au thin films as a function of film thickness.

The measured film stress is plotted in Figure 6. Previous studies reported that the residual stress strongly depended on the sputtering parameters, especially the chamber pressure [44,45,46]. In this experiment, the film stress was compressive for all film thicknesses, and the compressive residual stress decreased to −20 MPa when the film thickness was increased to 500 nm. Moreover, the change in the wafer bow after film deposition was less than 1 µm. This means that film stress and wafer bow due to residual stress in Au thin films should not affect bonding.

**Figure 7:**
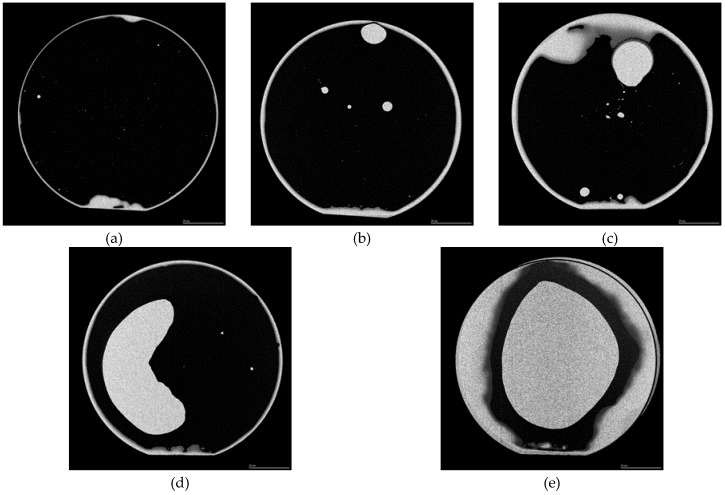
Typical surface acoustic microscope (SAM) images of room-temperature pressureless wafer-scale bonding with Au film thicknesses of (**a**–**e**) 15, 50, 100, 300, and 500 nm, respectively.

**Figure 8:**
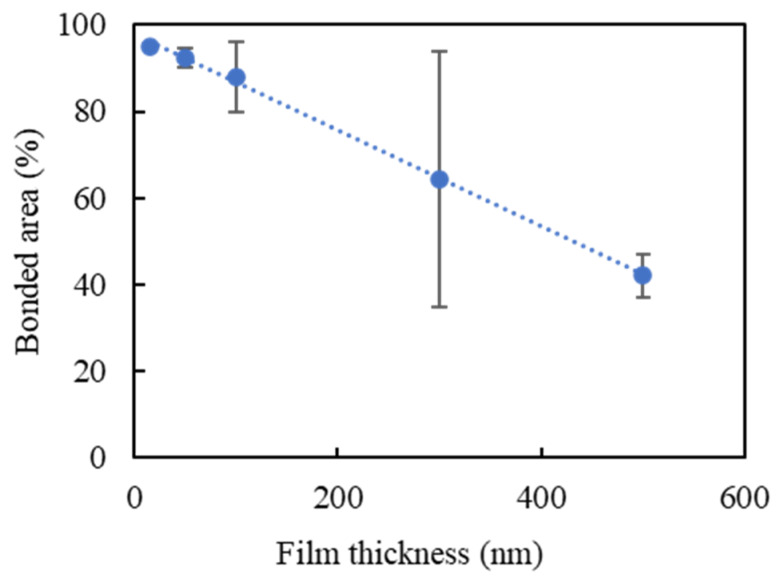
Relationship between bonded area and Au film thickness.

The bonded area of the room-temperature pressureless bonded wafers was observed with the SAM. Typical SAM measurement results are shown in Figure 7. Most of the wafer, except for the particles, was bonded successfully when Au thin films with a thickness of 100 nm or less were used. As shown in Figure 8, the bonded area was inversely proportional to the Au film thickness. When the film thickness was 15, 50, 100, or 300 nm, there was a sufficient bonding area for a razor blade test. Sufficient bonding strength over the surface energy of bulk Si (2.5 J/m^2^) [47] was obtained using Au thin films with a thickness of less than or equal to 300 nm although the entire wafer was not bonded when the thickness was 300 nm.

### 3.2. Room-Temperature Wafer Sealing in Vacuum

**Figure 9:**
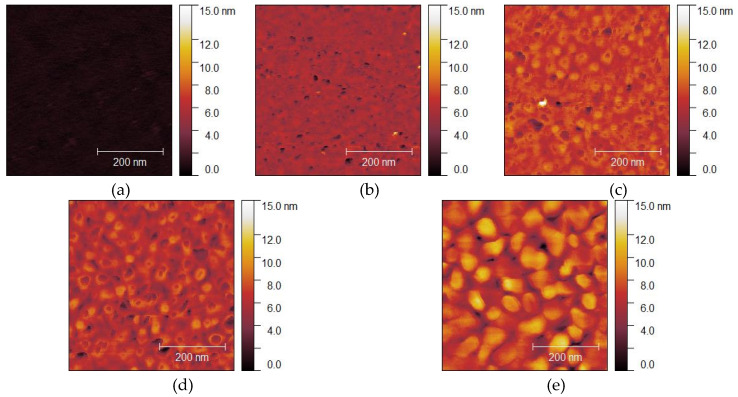
Typical AFM images of Au thin films deposited on Si wafers with cavities: (**a**) before deposition; (**b**–**e**) after deposition of films with thicknesses of 15, 50, 100, and 300 nm, respectively.

**Figure 10:**
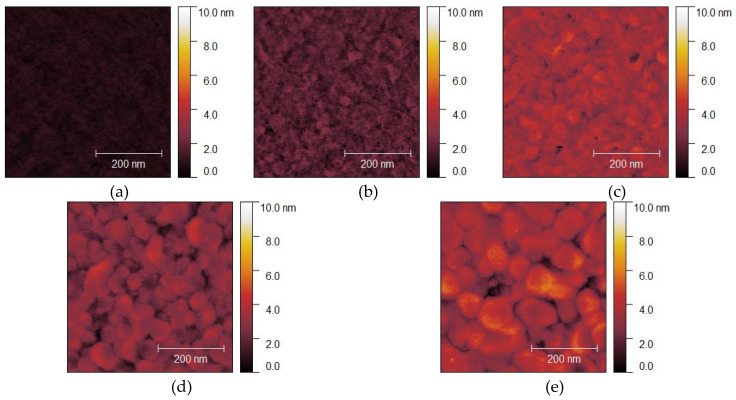
Typical AFM images of Au thin films deposited on glass wafers: (**a**) before deposition; (**b**–**e**) after deposition of films with thicknesses of 15, 50, 100, 300 nm, respectively.

**Figure 11:**
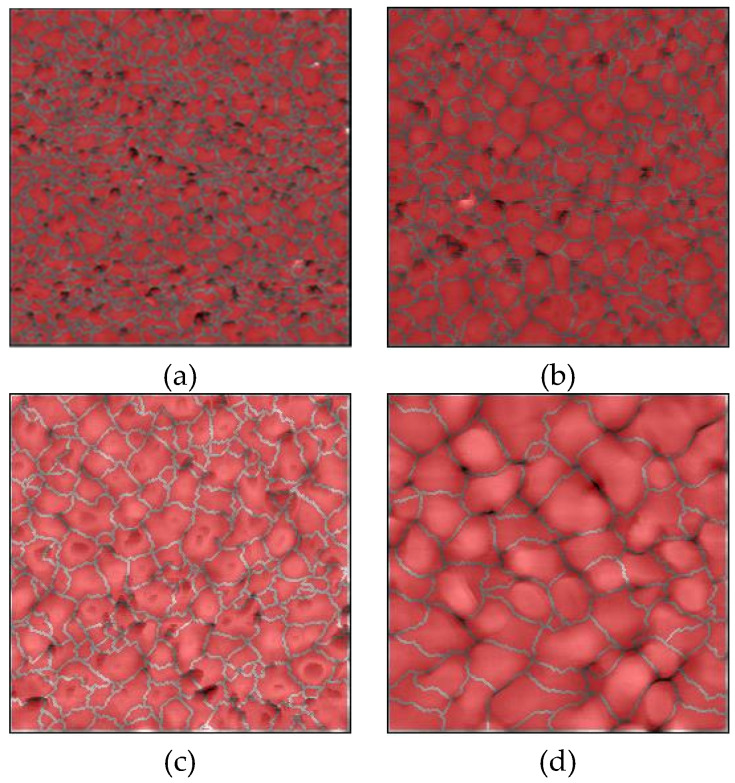
AFM images of Au thin films deposited on Si wafers with cavities with grains segmented using the watershed algorithm: (**a**–**d**) films with thicknesses of 15, 50, 100, 300 nm, respectively.

**Figure 12:**
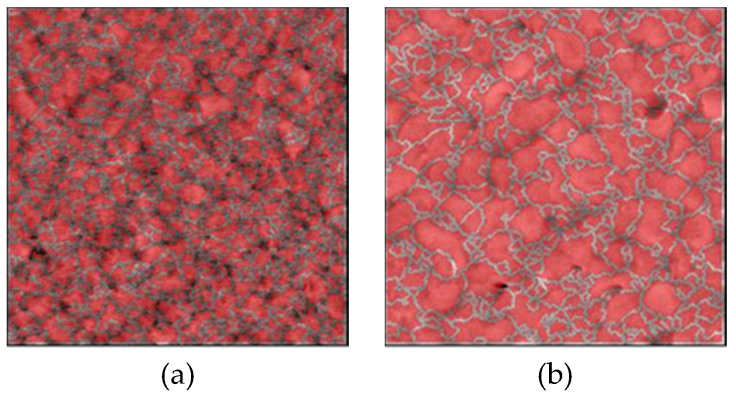

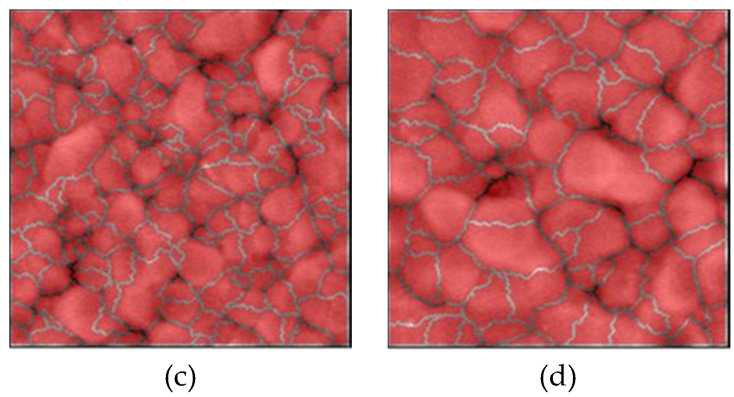
AFM images of Au thin films deposited on glass wafers with grains segmented using the watershed algorithm: (**a**–**d**) films with thicknesses of 15, 50, 100, 300 nm, respectively.

**Figure 13:**
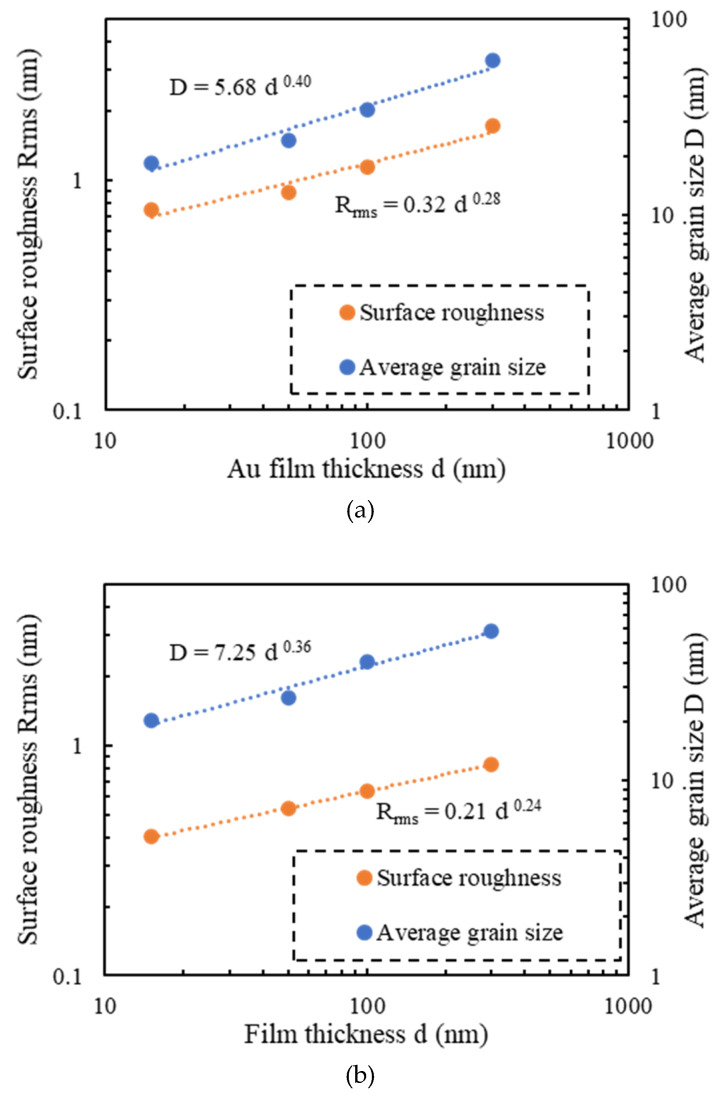
Effect of Au film thickness on surface roughness and average grain size: (**a**) films deposited on Si wafers with cavities; (**b**) films deposited on glass wafers.

Measured AFM images of Au thin films with thicknesses of 15, 50, 100, 300 nm deposited on Si wafers with cavities and glass wafers are shown in Figure 9 and Figure 10. The RMS surface roughness of the Si and glass wafers before film deposition was 0.2 nm. The AFM measurement results showed that the change in surface roughness due to wet chemical etching was small, and thus, had little effect on bonding. The grain geometry of each Au thin film was determined using the watershed algorithm. AFM images of Au thin films deposited on Si wafers with cavities with the grains segmented are shown in Figure 11 and Figure 12. The relationships between Au film thickness, surface roughness, and average grain size are plotted in Figure 13. The surface roughness and grain size increased exponentially as the film thickness was increased, which was consistent with the results when Au thin films were deposited on Si wafers (Figure 4). 

**Figure 14:**
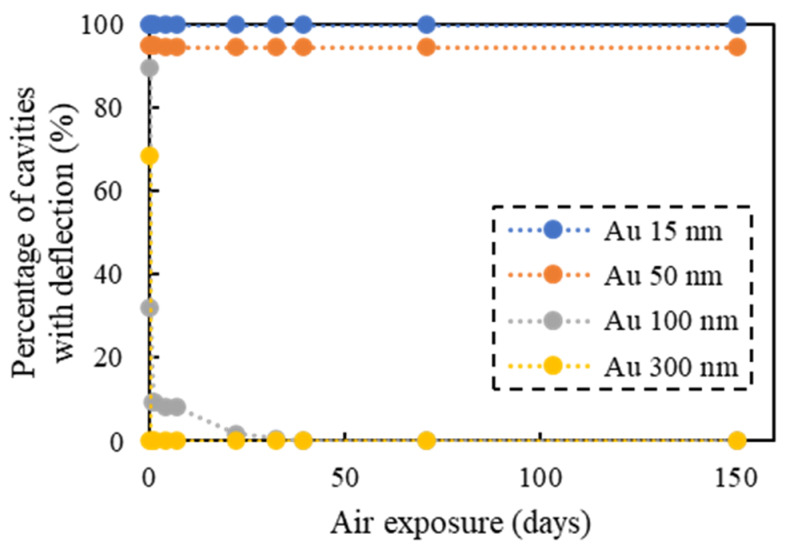
Vacuum sealing results: percentage of cavities with a deflection after air exposure.

The success or failure of the vacuum sealing was determined by observing the deflection of the glass caps caused by the differential pressure between the vacuum-sealed cavities and the ambient atmosphere. The measurement results are plotted in Figure 14. When the Au thin films were 100 nm thick or more, the deflection was observed only in the center region of the wafer immediately after bonding, and the deflection disappeared as the bonded wafer pairs were exposed to air. When the films were 50 nm thick or less, the deflection did not change even after 150 days of air exposure. These results indicated that Au thin films with a thickness of 50 nm or less could be used effectively for wafer bonding, especially for vacuum sealing.

Moreover, the air leakage of samples vacuum sealed using Au thin films with a thickness of 15 nm measured by the time dependence of the deflection of the thin glass caps was less than 1.3 × 10^−14^ Pa m^3^/s [35]. This satisfied the reject limit defined by MIL-STD-883 K, method 1014 (5.0 × 10^−9^ Pa m^3^/s).

**Figure 15:**
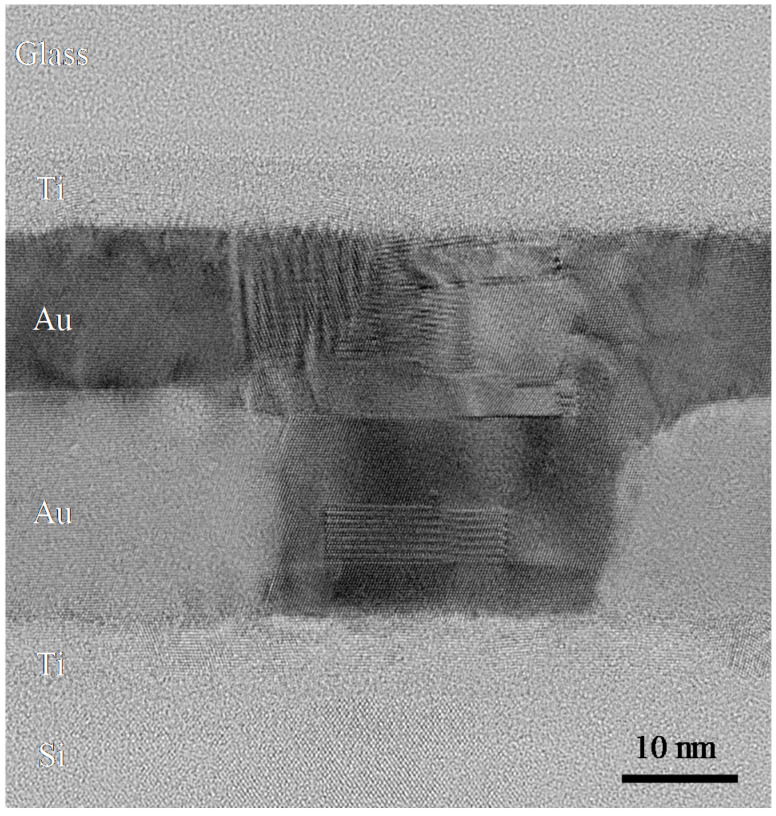
Transmission electron microscope (TEM) image of the bonded sample with a 15-nm-thick Au film.

Cross-sectional TEM observation of the Au-Au bonded interface with 15-nm-thick Au films was performed to investigate its microstructure. The example TEM image in Figure 15 shows that bonding was achieved at the atomic level and that Au atoms diffused around the grain boundaries. This indicated that good sealing could be obtained using 15-nm-thick Au films.

## 4. Conclusions

We investigated the effect of Au film thickness (15–500 nm) and surface roughness on room-temperature pressureless wafer bonding and sealing by Au-Au surface activated bonding. The RMS surface roughness and grain size of Au thin films sputtered on Si wafers, Si wafers with cavities, and glass wafers increased with the film thickness. When the film thickness was 100 nm or less, most of the wafer was bonded; the bonded area decreased as the Au film thickness was increased. Room-temperature wafer-scale vacuum sealing was achieved using Au thin films with a thickness of 50 nm or less. These results suggest that Au-Au surface activated bonding using ultrathin Au films is useful in achieving room-temperature wafer-level hermetic and vacuum packaging of MEMS and optoelectronic devices.
